# Accidental strangulation in children by the automatic closing of a car window

**DOI:** 10.5935/0103-507X.20180017

**Published:** 2018

**Authors:** Kailene Serena, Jefferson Pedro Piva, Cinara Andreolio, Paulo Roberto Antonacci Carvalho, Tais Sica da Rocha

**Affiliations:** 1 Pediatric Intensive Care Unit, Hospital das Clínicas de Porto Alegre, Universidade Federal do Rio Grande do Sul - Porto Alegre (RS), Brazil.; 2 Pediatrics, Universidade Federal do Rio Grande do Sul - Porto Alegre (RS), Brazil.

**Keywords:** Accident, Automobiles, Pulmonary edema, Child, Case reports

## Abstract

Among the main causes of death in our country are car accidents, drowning and accidental burns. Strangulation is a potentially fatal injury and an important cause of homicide and suicide among adults and adolescents. In children, its occurrence is usually accidental. However, in recent years, several cases of accidental strangulation in children around the world have been reported. A 2-year-old male patient was strangled in a car window. The patient was admitted to the pediatric intensive care unit with a Glasgow Coma Scale score of 8 and presented with progressive worsening of respiratory dysfunction and torpor. The patient also presented acute respiratory distress syndrome, acute pulmonary edema and shock. He was managed with protective mechanical ventilation, vasoactive drugs and antibiotic therapy. He was discharged from the intensive care unit without neurological or pulmonary sequelae. After 12 days of hospitalization, he was discharged from the hospital, and his state was very good. The incidence of automobile window strangulation is rare but of high morbidity and mortality due to the resulting choking mechanism. Fortunately, newer cars have devices that stop the automatic closing of the windows if resistance is encountered. However, considering the severity of complications strangulated patients experience, the intensive neuro-ventilatory and hemodynamic management of the pathologies involved is important to reduce morbidity and mortality, as is the need to implement new campaigns for the education of parents and caregivers of children, aiming to avoid easily preventable accidents and to optimize safety mechanisms in cars with electric windows.

## INTRODUCTION

Deaths related to external causes, such as car accidents, drowning and accidental burns, in pediatric and adolescent patients are frequent, especially in children under 1 year of age.^(1-3)^ Strangulation is a potentially fatal injury, and its occurrence in children is usually accidental. In recent years, several cases of accidental strangulation in children have been reported around the world.^(4)^

Strangulation in children is an uncommon but extremely serious and potentially fatal event due to its associated complications. After suffocation, there is a reduction in cerebral oxygenation, promoting varying degrees of edema, hemorrhage and ischemia.^(5)^ In cases of strangulation, the comparison between adults and children shows that they have a lower risk of laryngeal and bone fractures due to elasticity but are more susceptible to airway edema.^(5,6)^

In the literature, events associated with asphyxia, such as accidents in sleeping hammocks, the automatic closing of electric windows in cars and suicide attempts with ropes, among others, are described.^(7-10)^ Although the strangulation of children in stopped vehicles is poorly reported, this incident should be valued because it is easily avoidable and reflects the education given by parents or caregivers to children regarding their safety.

In this study, the authors discuss the management and treatment of children who are victims of strangulation caused by the automatic closing of automobile electric windows, evolving with severe respiratory compromise, pulmonary edema and acute respiratory distress syndrome (ARDS) but obtaining full recovery.

## CASE REPORT

**Figure 1:**
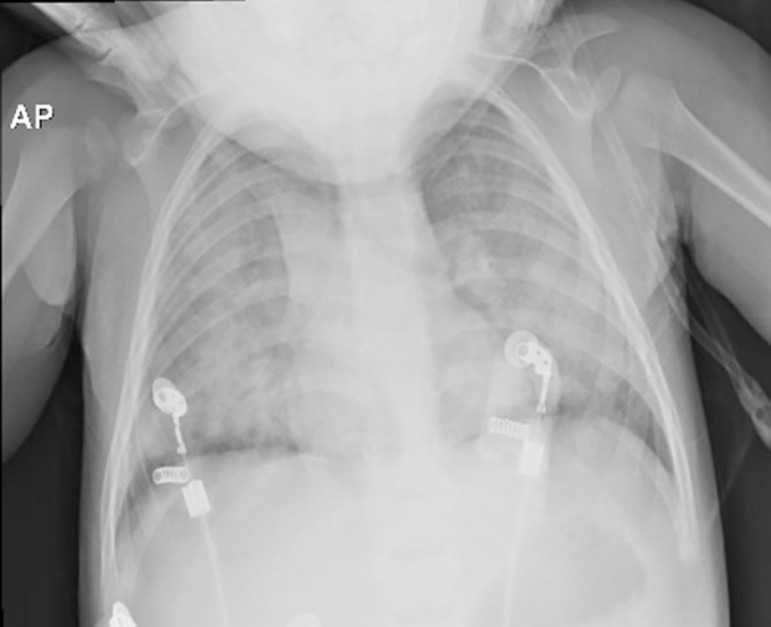
Chest X-ray at hospitalization (a few hours after the injury), in which pulmonary hypoexpansion with signs of alveolar-interstitial involvement suggestive of consolidations and pulmonary edema (i.e., acute respiratory distress syndrome) is observed.

A male patient, 2 years old, left unaccompanied inside a vehicle, was found unconscious with his head caught in the window of the car. The child was taken to an emergency room, where it was found that he had apnea, weak pulse, petechiae on the face and a hematoma in the cervical region. The patient was reanimated with positive pressure ventilation and resumed spontaneous ventilation within a few minutes. The patient underwent cranial and cervical tomography without changes and was transferred to the *Hospital das Clínicas de Porto Alegre*, where he arrived unconscious, with hematemesis and cyanosis of the extremities and oral mucosa. He was admitted to the pediatric intensive care unit (ICU) with a Glasgow Coma Scale score of 8, progressive worsening of torpor and respiratory dysfunction. Orotracheal intubation was performed, and bleeding was visualized through the tracheal tube. Under suspicion of an airway lesion due to local trauma, fibrobronchoscopy was performed, which did not reveal any structural alterations. A chest x-ray revealed bilateral pulmonary consolidations. The child's condition evolved with progressive worsening and refractory hypoxemia, requiring increases in the inspired oxygen fraction (FiO_2_) and ventilator parameters, with a diagnosis compatible with ARDS. Protective mechanical ventilation (MV) was performed, with positive end-respiratory pressure (PEEP) values between 12 and 16cmH_2_O, associated with FiO_2_ values between 0.4 and 0.7. The child required progressively higher doses of vasoactive drugs. On the following days, a positive cumulative fluid balance was observed when a continuous infusion of diuretic was administered. This measure, together with keeping the child in a prone position to manage the pulmonary ARDS, allowed the reduction of his MV parameters. Among the other treatments instituted were hypertonic solution instillation aimed at the prevention of cerebral edema, because there was hypoxic ischemic injury in the brain without a definite time frame, and antibiotic therapy for probable aspiration pneumonia. The MV and tracheal tube were suspended on the seventh day after the injury, and the patient was maintained on noninvasive ventilation for 24 hours immediately after extubation, with progressive improvement and discharge from the ICU in 10 days without neurological or pulmonary sequelae. After 12 days of hospital stay, he was discharged from the hospital in great condition (Figure 1).

## DISCUSSION

The occurrence of strangulation in children has increased considerably in recent years. There are several forms of strangulation, associated with automatic closing of electric windows in automobiles; suspension and hanging on sleeping hammocks, sarees (fabric used for women to cover the body) or ropes; and other mechanisms. In Western countries, strangulation is the third most common cause of accidental childhood deaths, 17% of which are caused by ropes and cables.^(9,10)^ Strangulation ranks fourth among the causes of unintentional injuries in children under 1 year of age, following road accidents, drowning and burns. The incidence of automobile window strangulation is rare but of high morbidity and mortality. Fortunately, newer cars have devices that stop the automatic closing of the windows when resistance is encountered.^(11,12)^

There have been reports of Indian children being suspended by the cervical region in homemade saree hammocks. In cases associated with longer times of strangulation, there was severe central nervous system impairment, long ICU stays and hospital times and discharge with severe sequelae.^(13)^

Considering the severity of asphyxia secondary to childhood strangulation, early identification and appropriate management of complications are of paramount importance. Brain damage and death are caused by airway obstruction, venous congestion leading to hypoxia, acidosis, bronchopneumonia, cerebral edema and irreversible damage to brain cells. Pulmonary complications such as aspiration pneumonia, bronchopneumonia and ARDS are important causes of morbidity and mortality.^(14,15)^ The prognosis depends on the severity and duration of the asphyxia. Mortality is high, and total neurological recovery has never been described in patients with described cardiac arrest associated with strangulation. The goal is to maintain adequate cerebral blood flow so that brain cells, in turn, maintain aerobic metabolism, even when the patient is in a deep coma.^(16)^

Cerebral edema results from alterations due to failures in intracellular ionic control, accumulation of sodium and water (cytotoxic edema) and control of flow and vascular permeability, leading to the accumulation of fluid in the cerebral interstice (vasogenic edema).^(17-19)^ Therefore, in these cases, one of the recommended initial therapeutic measures is to increase serum osmolarity from the first moments to balance the intra- and extracellular osmotic forces.^(20,21)^

In the case of strangulation reported by us, despite the sensory alterations present, demonstrating impairment of the central nervous system, cranial tomography still did not present the classic signs of cerebral edema and endocranial hypertension, namely ventricular reduction or collapse, disappearance of cortical sulci and diffuse edema, associated or not with hemorrhagic areas. This absence of suggestive classic images on tomography is not uncommon in the initial post-injury moments and should be interpreted as the phases of the establishment and progression of cytotoxic and vasogenic edema, which may culminate with intracranial hypertension (ICH).^(22)^ Early treatment of ICH aims to improve cerebral perfusion pressure, reduce the chances of herniation and improve neurological outcomes. Among the measures we emphasize are the early use of hypertonic solution for neuronal protection, with increased serum osmotic pressure (serum sodium between 155 - 165mEq/L);^(23)^ vasoactive drugs to optimize cerebral perfusion pressure, defined as a mean arterial pressure 50 - 60mmHg higher than the intracranial pressure (which in this case was estimated as 20mmHg); controlled MV; and pain/agitation control, with the use of continuous sedatives/analgesics.

The major osmotic agents for treating cerebral edema include mannitol and hypertonic saline. Mannitol is indicated in situations of acute hypertensive crises. However, because mannitol is an osmotic diuretic, it can cause hypotension and reduce cerebral perfusion. Hypertonic saline solution does not have the diuretic effect of mannitol; its mechanism involves increased serum osmolarity and consequent increases in the mean systemic blood pressure and cerebral perfusion pressure. It is now known that increases in sodium are not deleterious, as seen in the rapid correction of hyponatremia. In this situation, values close to 165mEq/L are safe and effective, without major adverse effects. We chose to start with an infusion of 0.1 - 1mL/kg/hour of 3% solution, reaching serum sodium concentrations between 150 - 165mEq/L.^(24)^

A frequent complication in these cases is the presence of aspiration pneumonia associated with ARDS. In this case, the dilemma of optimizing pulmonary treatment with increased intrathoracic pressures and, consequently, reducing venous drainage or using a less protective ventilation technique can be established to maximize venous return of the central nervous system. Some time ago, it was shown that the use of positive expiratory pressure (levels of 5, 10 and 15cmH_2_O) has little effect on the intracranial pressure of ICH patients.^(25,26)^ This finding can be explained by fluid mechanics, where a positive end-expiratory pressure (PEEP) of 13cmH_2_O equals a pressure of 10mmHg, which is lower than the pressure at which it is defined as ICH (> 20mmHg). Thus, even in the presence of 13 - 15cmH_2_O PEEP (inside the thorax), there would be venous flow in the central nervous system towards the thorax. Therefore, the use of PEEP (8 - 14cmH_2_O) allows the alveoli to remain open, improving oxygenation, reducing pulmonary vascular resistance and promoting better ventilation/perfusion ratios in patients with pulmonary lesions compatible with ARDS and cerebral edema.^(26-28)^

## CONCLUSION

In this report, the risk and severity of the complications of strangulated patients were evident. Their management should be oriented towards the optimization and preservation of hemodynamic, ventilatory and neurological functions. Efforts should be made to implement educational campaigns for parents and caregivers of children to avoid easily preventable accidents and to improve safety mechanisms in cars with electric windows.
